# Effect of prior mastectomy on outcomes following total shoulder arthroplasty

**DOI:** 10.1007/s00402-026-06215-5

**Published:** 2026-02-12

**Authors:** Akin Adio, Tarishi Parmar, Peter Boufadel, Hafiz F. Kassam, Adam Z. Khan, John G. Horneff, Brian W. Hill, Joseph A. Abboud

**Affiliations:** 1 University of Pennsylvania, Philadelphia, USA; 2 Rothman Orthopaedics, Philadelphia, USA; 3 Hoag Orthopedic Institute, Irvine, USA; 4 Southern California Permanente Medical Group, Panorama City, USA; 5 Palm Beach Orthopaedic Institute, Palm Beach Gardens, USA

**Keywords:** Mastectomy, Total shoulder arthroplasty, TriNetX, Breast cancer, VTE

## Abstract

**Background:**

Mastectomy and its associated treatments may alter shoulder biomechanics and soft-tissue integrity, yet their impact on total shoulder arthroplasty (TSA) outcomes remains unclear. This study evaluated short- and long-term complications following TSA in patients with a history of mastectomy.

**Methods:**

A retrospective cohort analysis was performed using the TriNetX Research Network, including patients who underwent primary TSA. Individuals with a history of mastectomy were identified using procedural and diagnostic coding, and those with contralateral mastectomy and TSA were excluded. Propensity score matching 1:1 was performed to balance demographics and comorbidities. Ninety-day and two-year outcomes were compared between matched cohorts.

**Results:**

After matching, 1,865 patients with prior mastectomy were compared with 1,865 controls. Patients with a history of mastectomy had a significantly higher incidence of postoperative lymphedema (3.48% vs. 0.67%; RR 5.15; *P* < .0001). They also demonstrated increased risk of venous thromboembolism (4.56% vs. 3.11%; RR 1.47; *P* = .018). Overall postoperative infection rates were higher in the mastectomy cohort (3.96% vs. 2.70%; RR 1.47; *P* = .0378), including higher rates of cellulitis (2.81% vs. 1.72%; RR 1.63; *P* = .031). No significant differences were observed for 2-year outcomes.

**Conclusion:**

Patients with a history of mastectomy undergoing TSA are at increased risk of perioperative postoperative lymphedema, thromboembolic events, and cellulitis. These findings highlight the importance of heightened perioperative surveillance.

**Supplementary Information:**

The online version contains supplementary material available at 10.1007/s00402-026-06215-5.

## Introduction

Total shoulder arthroplasty (TSA) is a well-established and effective surgical option for patients with advanced glenohumeral osteoarthritis, consistently shown to improve pain, function, and quality of life [1–4]. The demand for TSA is projected to rise exponentially over the coming decades [5]. As the number of TSAs increases, the patient population undergoing this procedure has become more diverse [6, 7]. Risk factors such as comorbidities, prior surgeries, and unique anatomical or physiological considerations can influence surgical outcomes and complication rates [8, 9]. Identifying vulnerable subgroups is therefore critical for guiding perioperative decision-making and patient counseling [10, 11]. 

One population of particular concern is breast cancer survivors who have undergone mastectomy. Breast cancer remains the most commonly diagnosed cancer among women worldwide, with over 4.1 million survivors currently living in the United States alone [12, 13]. Advances in screening and treatment have markedly improved survival rates, with five-year survival now exceeding 92% in early-stage disease [14]. Further, previous literature found that 72% of women diagnosed with early-stage breast cancer undergo mastectomy [15]. Consequently, a substantial population of these survivors may live long enough to develop degenerative joint conditions such as glenohumeral osteoarthritis. Short and long-term complications following mastectomy, however, are common and can have profound effects on shoulder health [16, 17]. Lymphedema, restricted shoulder range of motion, VTE, and weakness of the surrounding musculature are frequently reported sequelae [17–20]. Post-mastectomy patients also demonstrate impaired scapulothoracic kinematics, soft tissue scarring, and compromised chest wall mobility, which may increase mechanical strain on the glenohumeral joint [21, 22]. Notably, Andrews et al. reported outcomes in a small case series of 20 women, demonstrating meaningful pain relief but a high rate of soft-tissue–related complications, including lymphedema exacerbation and infection [23]. 

These factors raise concern that breast cancer survivors may represent a higher-risk group for adverse outcomes following TSA. Despite this, no large-scale study has directly evaluated whether a history of mastectomy increases postoperative complications in the setting of TSA, leaving surgeons without clear evidence to guide risk stratification and patient counseling. As such, the purpose of this study was to evaluate 90-day and 2-year postoperative complications and outcomes of TSA among patients with a history of mastectomy for breast cancer utilizing a multi-institutional database. We hypothesized that patients with a history of mastectomy undergoing total shoulder arthroplasty would be at increased risk for postoperative lymphedema, infection, and shoulder stiffness due to disrupted lymphatic drainage and regional soft-tissue compromise.

## Methods

### Data source

This retrospective cohort study evaluated short- and long-term complications following total shoulder arthroplasty in patients with a history of mastectomy. The analysis was performed using TriNetX, a database of deidentified electronic medical records aggregated from 63 health care organizations across the United States collaborative network. The query for patient data was executed on Jan 10, 2026. Because this study used only deidentified data and contained no individually identifiable information, Institutional Review Board approval was not required.

### Study population

Patients who underwent TSA were identified using standard Current Procedural Terminology (CPT) and International Classification of Diseases, Tenth Revision (ICD-10) procedure codes for total or reverse shoulder arthroplasty. Patients with a history of mastectomy within 5 years prior to TSA were identified using ICD-10 code Z90.1, encompassing acquired absence of the breast, and CPT codes 19301 through 19307, which include partial, simple, subcutaneous, modified radical, and radical mastectomy procedures. Individuals with axillary lymph node dissection were included. Patients with contralateral mastectomy and shoulder arthroplasty were excluded. Subgroup analyses for aromatase inhibitor use, radiation therapy, and lymphadenectomy were attempted but were underpowered due to small sample sizes.

### Propensity matching

Cohorts were propensity score–matched 1:1 using logistic regression on the TriNetX platform with nearest-neighbor matching, ensuring that the differences between variables exceed *P* =.05 for all covariates after matching. Matching variables included demographic characteristics and relevant clinical factors such as body mass index, diabetes mellitus, hypertension, hyperlipidemia, chronic kidney disease, liver disease, osteoporosis, tobacco use, alcohol use disorder, and chronic corticosteroid use.

### Outcomes

The primary outcomes included lymphedema, wound dehiscence, sepsis, venous thromboembolism (VTE), acute kidney injury (AKI), and nerve injury within 90 days following TSA. Infectious outcomes were also evaluated at 90 days and included overall infection, surgical site infection, cellulitis, erysipelas, periprosthetic joint infection, and deep infection. Two-year outcomes included mechanical dislocation, periprosthetic fracture, loosening, shoulder instability, periprosthetic joint infection (PJI), shoulder stiffness, and revision TSA.

### Statistical analysis

Relative risks (RRs), 95% confidence intervals (CIs), and p-values were computed using the TriNetX analytics platform. Categorical variables were compared using chi-squared tests, and continuous variables were analyzed using Student’s *t*-tests. Statistical significance was defined as *p* <.05 (bolded in tables).

## Results

### Cohort characteristics

The query identified 1,868 patients had a documented history of mastectomy within 5 years prior to TSA, while 62,170 had no history of mastectomy. Before matching, patients with prior mastectomy were significantly older, more often Hispanic, and had greater rates of hypertension, hyperlipidemia, chronic ischemic heart disease, heart failure, atherosclerosis, chronic obstructive pulmonary disease, chronic kidney disease, liver disease, diabetes mellitus, obesity, anemia, disorders of bone density, alcohol-related disorders, venous thromboembolism, and higher BMI. After 1:1 propensity score matching, each cohort consisted of 1,865 patients, and there were no statistically significant differences in baseline demographic or clinical characteristics between the two groups. (Table 1)

**Table 1:** Comparison of demographic characteristics between mastectomy and non-mastectomy cohorts

Characteristics	Cohort	Before matching			After matching		
		Patients	% Patients	*P*-value	Patients	% Patients	*P*-value
Demographics							
Age at index	Mastectomy	1868 (73.1 ± 9.04)	100%	**< 0.0001**	1865 (73.1 ± 9.04)	100%	0.5868
	No Mastectomy	62,170 (69.5 ± 10.5)	100%		1865 (72.9 ± 8.86)	100%	
White	Mastectomy	1619	86.67%	0.0648	1617	86.702%	0.2178
	No Mastectomy	52,925	85.129%		1642	88.043%	
Not hispanic or latino	Mastectomy	1570	84.047%	**< 0.0001**	1567	84.021%	0.7192
	No Mastectomy	48,561	78.11%		1575	84.45%	
Black or African American	Mastectomy	170	9.101%	0.68	169	9.062%	0.2660
	No Mastectomy	5487	8.826%		150	8.043%	
Comorbidities							
Hypertensive diseases	Mastectomy	1442	77.195%	**< 0.0001**	1,439	77.158%	0.3650
	No Mastectomy	36,336	58.446%		1,462	78.391%	
Hyperlipidemia	Mastectomy	1063	56.906%	**< 0.0001**	1060	56.836%	0.6671
	No Mastectomy	24,511	39.426%		1073	57.534%	
Disorders of bone density and structure	Mastectomy	947	50.696%	**< 0.0001**	944	50.617%	0.9217
	No Mastectomy	18,153	29.199%		941	50.456%	
Overweight and obesity	Mastectomy	756	40.471%	**< 0.0001**	753	40.375%	0.8414
	No Mastectomy	16,356	26.309%		759	40.697%	
Anemia	Mastectomy	563	30.139%	**< 0.0001**	560	30.027%	0.6952
	No Mastectomy	11,001	17.695%		571	30.617%	
Diabetes mellitus	Mastectomy	558	29.872%	**< 0.0001**	556	29.812%	0.8018
	No Mastectomy	13,351	21.475%		549	29.437%	
Ischemic heart diseases	Mastectomy	438	23.448%	**< 0.0001**	435	23.324%	0.1692
	No Mastectomy	8933	14.369%		400	21.448%	
Heart failure	Mastectomy	316	16.916%	**< 0.0001**	313	16.783%	0.1240
	No Mastectomy	4984	8.017%		258	13.834%	
Chronic kidney disease	Mastectomy	300	16.06%	**< 0.0001**	300	16.086%	0.1448
	No Mastectomy	6585	10.592%		268	14.37%	
Chronic obstructive pulmonary disease	Mastectomy	288	15.418%	**< 0.0001**	287	15.389%	0.1136
	No Mastectomy	7063	11.361%		253	13.566%	
Liver disease	Mastectomy	290	15.525%	**< 0.0001**	287	15.389%	0.7181
	No Mastectomy	5141	8.269%		295	15.818%	
Atherosclerosis	Mastectomy	215	11.51%	**< 0.0001**	212	11.367%	0.7555
	No Mastectomy	3379	5.435%		206	11.046%	
Nicotine dependence	Mastectomy	196	10.493%	0.3755	196	10.509%	0.6273
	No Mastectomy	6,137	9.871%		187	10.027%	
Venous thromboembolism	Mastectomy	167	8.94%	**< 0.0001**	164	8.794%	0.8629
	No Mastectomy	2789	4.486%		167	8.954%	
Alchohol substance abuse	Mastectomy	74	3.961%	**0.0136**	74	3.968%	0.3383
	No Mastectomy	1848	2.972%		63	3.378%	
Tobacco use	Mastectomy	59	3.158%	0.7345	59	3.164%	0.1082
	No Mastectomy	2,052	3.301%		43	2.306%	
Laboratory							
BMI	Mastectomy	1520 (30.8 ± 7.22)	81.37%	0.272	1517 (30.8 ± 7.22)	81.34%	0.0893
	No Mastectomy	44,913 (31 ± 7.48)	72.242%		1518 (31.2 ± 7.43)	81.394%	

### 90-day complications

Patients with a history of mastectomy had significantly higher rates of postoperative lymphedema (3.48% versus 0.67%, RR 5.15, *P* <.0001) and venous thromboembolism (4.56% versus 3.11%, RR 1.47, *P* =.018) compared with those without mastectomy. Infectious complications within 90 days were also more frequent in the mastectomy cohort (3.96% vs. 2.70%, RR 1.47, *P* =.037), driven primarily by higher rates of cellulitis (2.81% vs. 1.72%; RR 1.63; *P* =.031).There were no significant differences between groups for sepsis (*P* =.449), wound dehiscence (*P* = 1.00), nerve injury (*P* =.629), or acute kidney injury (*P* =.864). Rates of 90-day postoperative complications following TSA in patients with and without a history of mastectomy are presented in Table 2.

**Table 2:** Analysis of 90-day postoperative medical complications in total shoulder arthroplasty patients

Mastectomy vs. no mastectomy (*n* = 1865)					
Outcome	Incidence (%)		RR	95% CI	*P*-value
	Mastectomy	No mastectomy			
Lymphedema	3.48%	0.67%	5.15	(2.85–9.30)	**< 0.0001**
Sepsis	1.82%	1.50%	1.2	(0.74–1.97)	0.449
Wound dehiscence	0.58%	0.58%	1	(0.56–1.78)	1.000
Venous thromboembolism	4.56%	3.11%	1.47	(1.06–2.02)	**0.018**
Nerve injury	1.09%	0.93%	1.17	(0.62–2.18)	0.629
AKI	3.73%	3.63%	1.03	(0.75–1.42)	0.864
Total infection	3.96%	2.70%	1.47	(1.02–2.11)	**0.038**
SSI	0.92%	0.57%	1.6	(0.73–3.52)	0.237
Cellulitis	2.81%	1.72%	1.63	(1.04–2.56)	**0.031**
Erysipela	0.00%	0.00%	N/A	N/A	N/A
PJI	0.00%	0.00%	N/A	N/A	N/A
Deep infection*	N/A	N/A	N/A	N/A	N/A

*TriNetX masks patient outcomes with counts <10 for patient anonymity

### 2-year complications

At two years following TSA, there were no significant differences between patients with and without a history of mastectomy in rates of all mechanical complications (8.41% versus 8.74%, RR 0.96, *P* =.599), mechanical dislocation (1.54% versus 1.72%, RR 0.89, *P* =.531), loosening (0.95% versus 1.21%, RR 0.79, *P* =.273), periprosthetic fracture (1.95% versus 1.46%, RR 1.33, *P* =.175), shoulder instability (2.82% versus 2.95%, RR 0.96, *P* =.735), periprosthetic joint infection (1.42% versus 1.04%, RR 1.37, *P* =.288), postoperative shoulder stiffness (8.20% versus 7.35%, RR 1.12, *P* =.328), or revision surgery (1.85% versus 1.74%, RR 1.06, *P* =.733). Rates of 2-year postoperative complications following TSA in patients with and without a history of prior mastectomy are presented in Table 3.

**Table 3:** Analysis of 2-year postoperative medical complications in total shoulder arthroplasty patients

Mastectomy vs. no mastectomy (*n* = 1865)					
Outcome	Incidence (%)		RR	CI	*P*-value
	Mastectomy	No mastectomy			
All mechanical complications	8.41%	8.74%	0.96	(0.83–1.11)	0.599
Mechanical dislocation	1.54%	1.72%	0.89	(0.63–1.27)	0.531
Loosening	0.95%	1.21%	0.79	(0.51–1.21)	0.272
Periprosthetic fracture	1.95%	1.46%	1.33	(0.86–2.25)	0.1751
Shoulder Instability	2.82%	2.95%	0.96	(0.74–1.24)	0.7352
Periprosthetic joint infection	1.42%	1.04%	1.37	(0.76–2.47)	0.2878
Shoulder stiffness	8.20%	7.35%	1.12	0.89–1.39	0.3279
Revision	1.85%	1.74%	1.06	(0.76–1.47)	0.733

## Discussion

In this large TriNetX database analysis of patients undergoing total shoulder arthroplasty, those with a history of mastectomy demonstrated significantly higher rates of early postoperative lymphedema, venous thromboembolism, and cellulitis compared with patients without mastectomy. However, no significant differences were observed in mechanical complications, implant failure, stiffness, or revision surgery at two years, indicating that long-term mechanical outcomes following TSA are comparable in this population.

At 90 days following TSA, lymphedema incidence was markedly higher in patients with a prior mastectomy. Similarly, Andrews et al. reported high rates of postoperative lymphedema in patients undergoing shoulder arthroplasty after mastectomy. Mastectomy with axillary lymph node dissection is a major cause of secondary lymphedema, occurring in up to 20–40% of breast cancer patients [24]. Studies demonstrate that chronic lymphatic injury following mastectomy leads to long-term impairment of lymphatic transport capacity, which can be reactivated or exacerbated by subsequent surgical trauma, infection, or venous congestion [25, 26]. Moreover, prospective data by Warren et al. confirm that regional lymph node radiation, which is commonly used along with mastectomy, markedly increases the long-term risk of lymphedema [27]. In their cohort of 1,476 breast cancer patients, the 2-year cumulative incidence was 21–22% among those receiving supraclavicular or axillary boost radiotherapy, compared with only 3% for those without regional nodal radiation. This chronic vulnerability means that patients with prior mastectomy are at elevated risk of postoperative limb swelling and lymphedema when undergoing later procedures. It is also clinically significant given the potential for lymphedema to worsen infection risk, delay recovery, and impair functional outcomes after surgery [28]. 

Cellulitis was significantly higher post-TSA among mastectomy patients. Prior research shows that chronic lymphedema predisposes to recurrent infection due to impaired local immune surveillance and lymphatic drainage [29–31]. Similarly, infection risk is elevated in patients with chronic lymphedema, as both impair soft tissue healing and immune response around prosthetic implants [28, 32]. ^,^ [33, 34] Because rates of wound dehiscence were low in this cohort, our ability to evaluate how prior soft-tissue disruption may contribute to postoperative infection risk in patients is limited. Nonetheless, these results highlight the need for careful infection prophylaxis and soft-tissue management in post-mastectomy TSA patients, particularly those with lymphedema and prior radiation exposure.

We found elevated VTE rates in the mastectomy cohort. While mastectomy itself is not independently associated with long-term hypercoagulability, cancer and adjuvant therapies (particularly chemotherapy and hormonal treatments) significantly increase VTE risk [35, 36]. Further, given that VTE is common in cancer in general, the elevated rates observed here likely reflect residual prothrombotic effects of prior cancer therapy rather than the mastectomy procedure itself [37]. To date, limited data exist on whether mastectomy alone increases postoperative PE risk in later surgeries such as TSA, making this a potential area for further study. Nonetheless, our findings underscore the importance of perioperative thromboprophylaxis and individualized VTE risk assessment in this patient population.

We hypothesized that prior mastectomy and potential associated oncologic therapies would increase the risk of stiffness after TSA. Women undergoing mastectomy are more than three times as likely to report shoulder stiffness compared with those undergoing breast-conserving surgery [20, 38]. This is due to due to regional soft tissue compromise and altered scapulohumeral biomechanics [39]. However, we did not observe significant differences. This finding may reflect limitations of administrative coding, which may fail to capture postoperative stiffness. Future studies incorporating patient-reported outcome measures and functional scores are needed to better define the clinical impact of mastectomy on stiffness after shoulder arthroplasty.

To our knowledge, this is the first large-scale, population-based analysis to evaluate the impact of mastectomy on total shoulder arthroplasty. However, there are several limitations. First, as a retrospective study using the TriNetX database, our findings are susceptible to selection bias and variation in coding accuracy across electronic medical record systems [40]. Although propensity matching reduced baseline differences, unmeasured confounders such as operative technique, implant selection, and rehabilitation may still influence outcomes. Secondly, we attempted subgroup analyses examining patients with history aromatase inhibitor exposure, radiation, and lymph node resection. However, these analyses showed insignificant results, likely underpowered due to sample size limitations. Additionally, the observed increase in lymphedema risk may be influenced by increased surveillance and coding practices in patients with prior mastectomy [23]. Another limitation is that the database does not distinguish between anatomic and reverse arthroplasty, which may differ in their postoperative complication profiles and outcomes. Furthermore, recurrence of breast cancer and adjuvant therapies factors that may influence two-year complication rates were not assessed. Future studies with larger cohorts could examine these comparisons examining how treatment related factors and mastectomy subtypes influence soft-tissue healing, bone quality, and infection risk in shoulder arthroplasty.

## Conclusion

Patients with a history of mastectomy undergoing total shoulder arthroplasty experienced higher rates of early postoperative lymphedema, thromboembolic events, and cellulitis compared with those without mastectomy, while no significant differences were observed in mechanical complications, stiffness, or revision surgery at two years. These findings suggest that cancer-related alterations in lymphatic drainage and soft-tissue integrity contribute primarily to elevated perioperative medical risk rather than long-term mechanical failure. Recognition of these risks and implementation of targeted perioperative surveillance and prophylactic strategies may improve outcomes in this population.

## Supplementary Information

Below is the link to the electronic supplementary material.

Supplementary Material 1

## Data Availability

The data that support the findings of this study were obtained from the TriNetX Research Network. Restrictions apply to the availability of these data, which were used under license for the current study and are not publicly available. However, data may be made available from the corresponding author upon reasonable request and with the permission of TriNetX.
