# Supplementary material for: Effect of prior mastectomy on outcomes following total shoulder arthroplasty
Source: Arch Orthop Trauma Surg. 2026 Feb 12;146(1):62. doi: 10.1007/s00402-026-06215-5 (PMC12901221; doi:10.1007/s00402-026-06215-5)
Supplement: Supplementary file 1 — Supplementary Material 1 [file 402_2026_6215_MOESM1_ESM.docx]

| **Variable** | **Description** | **Code** |
| --- | --- | --- |
| Wound Dehiscence | Disruption of a wound, not elsewhere classified | ICD10CM:T81.3 |
| VTE | Acute embolism and thrombosis of deep veins of lower extremity; or Acute embolism and thrombosis of unspecified deep veins of lower extremity; or Acute embolism and thrombosis of unspecified deep veins of unspecified lower extremity + Pulmonary embolism. | ICD10CM:I26, ICD10CM:I82.4 |
| Lymphedema | Lymphedema, not elsewhere classified | ICD10CM:I89.0 |
| Nerve Injury | Injury of brachial plexus | ICD10CM:S14.3 |
| Acute Kidney Injury | Unspecified kidney failure; or Acute kidney failure. | ICD10CM:N19; ICD10CM:N17 |
| SSI | Infection Following a Procedure | ICD10CM: T81.41 |
| Cellulitis | Cellulitis of right and left upper limb | ICD10CM: L03.113, L03.114 |
| Deep Infection | Infection following a procedure, deep and organ incisional surgical site | ICD10CM: T81.42, T81.43 |
| Erysipela | Erysipelas | ICD10CM: A46 |
| Sepsis | Sepsis, unspecified | ICD10CM: A41 |
| Periprosthetic joint infection (PJI), | Infection and inflammatory reaction due to other internal joint prosthesis | ICD10CM:T84.59 |
| 2 year |  |  |
| Revision | Revision of total shoulder arthroplasty, including allograft when performed; humeral and/or glenoid component | CPT: 23473; 23474 |
| Periprosthetic Joint Infection | Infection and inflammatory reaction due to other internal joint prosthesis. | ICD10CM:T84.59 |
| Mechanical Loosening | Mechanical loosening of other internal prosthetic joint. | ICD10CM:T84.038 |
| Dislocation | Dislocation of unspecified internal joint prosthesis; or Dislocation of other internal joint prosthesis. | ICD10CM:T84.029; ICD10CM:T84.028 |
| Shoulder Instability | Subluxation and dislocation of the shoulder joint | ICD10CM:M25.31 |
| Periprosthetic Fracture | Periprosthetic fracture around internal prosthetic shoulder joint | ICD10CM:M97.3 |
